# *N*-(2-Methoxy­phen­yl)phthalimide

**DOI:** 10.1107/S1600536809032826

**Published:** 2009-08-22

**Authors:** Yoke Ling Sim, Azhar Ariffin, Mohammad Niyaz Khan, Seik Weng Ng

**Affiliations:** a Department of Chemistry, University of Malaya, 50603 Kuala Lumpur, Malaysia

## Abstract

The phthalimide fused-ring system and the phenyl­ene ring in the title compound, C_15_H_11_NO_3_, are inclined at an angle of 54.2 (1)°.

## Related literature

For the crystal structures of *N*-(phen­yl)phthalimides, see: Izotova *et al.* (2009 ▶); Magomedova *et al.* (1980 ▶). For that of the 2-ethyl-substituted derivative, see: Fan *et al.* (2008 ▶).
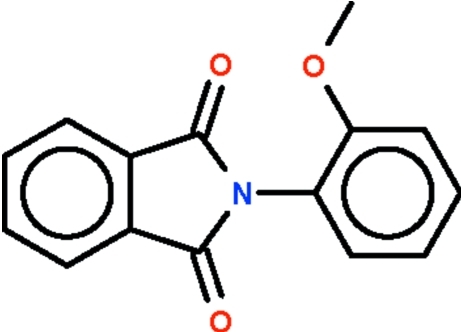


## Experimental

### 

#### Crystal data

C_15_H_11_NO_3_
*M* *_r_* = 253.25
Monoclinic, 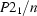

*a* = 11.8505 (2) Å
*b* = 6.6903 (1) Å
*c* = 15.3264 (3) Å
β = 106.258 (1)°
*V* = 1166.54 (3) Å^3^
*Z* = 4
Mo *K*α radiation
μ = 0.10 mm^−1^
*T* = 123 K
0.28 × 0.16 × 0.04 mm

#### Data collection

Bruker SMART APEX diffractometer
Absorption correction: none
10672 measured reflections
2682 independent reflections
2190 reflections with *I* > 2σ(*I*)
*R*_int_ = 0.028

#### Refinement

*R*[*F* ^2^ > 2σ(*F* ^2^)] = 0.036
*wR*(*F* ^2^) = 0.098
*S* = 1.03
2682 reflections
173 parameters
H-atom parameters constrained
Δρ_max_ = 0.27 e Å^−3^
Δρ_min_ = −0.22 e Å^−3^

### 

Data collection: *APEX2* (Bruker, 2008 ▶); cell refinement: *SAINT* (Bruker, 2008 ▶); data reduction: *SAINT*; program(s) used to solve structure: *SHELXS97* (Sheldrick, 2008 ▶); program(s) used to refine structure: *SHELXL97* (Sheldrick, 2008 ▶); molecular graphics: *X-SEED* (Barbour, 2001 ▶); software used to prepare material for publication: *publCIF* (Westrip, 2009 ▶).

## Supplementary Material

Crystal structure: contains datablocks global, I. DOI: 10.1107/S1600536809032826/bt5039sup1.cif

Structure factors: contains datablocks I. DOI: 10.1107/S1600536809032826/bt5039Isup2.hkl

Additional supplementary materials:  crystallographic information; 3D view; checkCIF report

## Figures and Tables

**Fig. 1.**
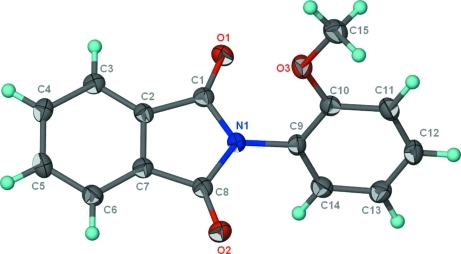
Anisotropic displacement ellipsoid plot (Barbour, 2001) of C15H11NO3 at the 70% probability level; hydrogen atoms are drawn as spheres of arbitrary radius.

## supplementary crystallographic information

### Experimental

Phthalic anhydride (5.00 g, 33.8 mmol) and 4-methoxyaniline (4.99 g, 40.5 mmol) were heated in acetic acid (15 ml) for 4 h. The mixture was cooled and then poured into water. The solid that separated was collected and recrystallized from ethanol in 90% yield.

### Refinement

H-atoms were placed in calculated positions (C—H 0.95 or 0.98 Å) and were included in the refinement in the riding model approximation, with *U*(H) set to 1.2 *U_eq_*(C) or 1.5*U_eq_*(C_methyl_).

### Crystal data

**Table:**

C_15_H_11_NO_3_	*F*(000) = 528
*M**_r_* = 253.25	*D*_x_ = 1.442 Mg m^−^^3^
Monoclinic, *P*2_1_/*n*	Mo *K*α radiation, λ = 0.71073 Å
Hall symbol: -P 2yn	Cell parameters from 3112 reflections
*a* = 11.8505 (2) Å	θ = 2.5–28.3°
*b* = 6.6903 (1) Å	µ = 0.10 mm^−^^1^
*c* = 15.3264 (3) Å	*T* = 123 K
β = 106.258 (1)°	Irregular, colorless
*V* = 1166.54 (3) Å^3^	0.28 × 0.16 × 0.04 mm
*Z* = 4	

### Data collection

**Table:**

Bruker SMART APEX diffractometer	2190 reflections with *I* > 2σ(*I*)
Radiation source: fine-focus sealed tube	*R*_int_ = 0.028
graphite	θ_max_ = 27.5°, θ_min_ = 1.9°
ω scans	*h* = −14→15
10672 measured reflections	*k* = −8→8
2682 independent reflections	*l* = −19→19

### Refinement

**Table:**

Refinement on *F*^2^	Primary atom site location: structure-invariant direct methods
Least-squares matrix: full	Secondary atom site location: difference Fourier map
*R*[*F*^2^ > 2σ(*F*^2^)] = 0.036	Hydrogen site location: inferred from neighbouring sites
*wR*(*F*^2^) = 0.098	H-atom parameters constrained
*S* = 1.03	*w* = 1/[σ^2^(*F*_o_^2^) + (0.0506*P*)^2^ + 0.3213*P*] where *P* = (*F*_o_^2^ + 2*F*_c_^2^)/3
2682 reflections	(Δ/σ)_max_ = 0.001
173 parameters	Δρ_max_ = 0.27 e Å^−^^3^
0 restraints	Δρ_min_ = −0.22 e Å^−^^3^

### Fractional atomic coordinates and isotropic or equivalent isotropic displacement parameters (Å^2^)

**Table:**

	*x*	*y*	*z*	*U*_iso_*/*U*_eq_	
O1	0.76144 (8)	0.26041 (14)	0.47272 (6)	0.0232 (2)	
O2	0.74087 (8)	0.35172 (14)	0.76449 (6)	0.0242 (2)	
O3	0.89049 (8)	0.63238 (14)	0.56474 (6)	0.0240 (2)	
N1	0.78134 (9)	0.32357 (16)	0.62507 (7)	0.0183 (2)	
C1	0.71795 (11)	0.28292 (18)	0.53464 (8)	0.0177 (3)	
C2	0.59283 (11)	0.26801 (17)	0.53474 (8)	0.0168 (3)	
C3	0.49248 (11)	0.23766 (18)	0.46394 (9)	0.0187 (3)	
H3	0.4962	0.2240	0.4031	0.022*	
C4	0.38571 (11)	0.22777 (19)	0.48501 (9)	0.0205 (3)	
H4	0.3149	0.2111	0.4376	0.025*	
C5	0.38127 (11)	0.24202 (19)	0.57471 (9)	0.0227 (3)	
H5	0.3075	0.2333	0.5875	0.027*	
C6	0.48287 (11)	0.26884 (19)	0.64603 (9)	0.0203 (3)	
H6	0.4801	0.2758	0.7073	0.024*	
C7	0.58786 (11)	0.28491 (18)	0.62405 (8)	0.0178 (3)	
C8	0.70848 (11)	0.32285 (18)	0.68350 (8)	0.0180 (3)	
C9	0.90625 (10)	0.34589 (19)	0.65589 (8)	0.0183 (3)	
C10	0.96165 (11)	0.50402 (19)	0.62429 (8)	0.0187 (3)	
C11	1.08374 (11)	0.5197 (2)	0.65517 (8)	0.0226 (3)	
H11	1.1233	0.6222	0.6324	0.027*	
C12	1.14739 (12)	0.3852 (2)	0.71924 (9)	0.0251 (3)	
H12	1.2304	0.3983	0.7409	0.030*	
C13	1.09199 (12)	0.2325 (2)	0.75213 (9)	0.0242 (3)	
H13	1.1362	0.1429	0.7968	0.029*	
C14	0.97095 (11)	0.2121 (2)	0.71899 (9)	0.0217 (3)	
H14	0.9323	0.1054	0.7398	0.026*	
C15	0.94409 (12)	0.8082 (2)	0.54130 (10)	0.0254 (3)	
H15A	0.9847	0.8806	0.5968	0.038*	
H15B	0.8835	0.8942	0.5026	0.038*	
H15C	1.0009	0.7703	0.5084	0.038*	

### Atomic displacement parameters (Å^2^)

**Table:**

	*U*^11^	*U*^22^	*U*^33^	*U*^12^	*U*^13^	*U*^23^
O1	0.0218 (5)	0.0299 (5)	0.0200 (5)	−0.0019 (4)	0.0091 (4)	−0.0012 (4)
O2	0.0228 (5)	0.0319 (5)	0.0176 (5)	−0.0004 (4)	0.0051 (4)	−0.0019 (4)
O3	0.0189 (5)	0.0231 (5)	0.0292 (5)	−0.0019 (4)	0.0052 (4)	0.0068 (4)
N1	0.0151 (5)	0.0223 (6)	0.0175 (5)	−0.0013 (4)	0.0047 (4)	0.0012 (4)
C1	0.0196 (6)	0.0152 (6)	0.0183 (6)	−0.0002 (4)	0.0054 (5)	0.0018 (5)
C2	0.0174 (6)	0.0138 (6)	0.0199 (6)	0.0000 (4)	0.0066 (5)	0.0019 (4)
C3	0.0211 (6)	0.0160 (6)	0.0187 (6)	0.0010 (5)	0.0049 (5)	0.0006 (5)
C4	0.0173 (6)	0.0180 (6)	0.0240 (6)	0.0000 (5)	0.0019 (5)	0.0002 (5)
C5	0.0176 (6)	0.0235 (7)	0.0282 (7)	−0.0013 (5)	0.0084 (5)	−0.0013 (5)
C6	0.0198 (6)	0.0220 (7)	0.0205 (6)	−0.0007 (5)	0.0079 (5)	−0.0001 (5)
C7	0.0196 (6)	0.0151 (6)	0.0186 (6)	−0.0006 (5)	0.0053 (5)	0.0005 (5)
C8	0.0182 (6)	0.0161 (6)	0.0201 (6)	0.0010 (5)	0.0063 (5)	0.0017 (5)
C9	0.0148 (6)	0.0226 (6)	0.0177 (6)	−0.0009 (5)	0.0047 (5)	−0.0025 (5)
C10	0.0186 (6)	0.0209 (6)	0.0174 (6)	0.0003 (5)	0.0061 (5)	−0.0016 (5)
C11	0.0193 (6)	0.0259 (7)	0.0242 (6)	−0.0044 (5)	0.0089 (5)	−0.0016 (5)
C12	0.0155 (6)	0.0337 (8)	0.0256 (7)	−0.0005 (5)	0.0050 (5)	−0.0022 (6)
C13	0.0206 (7)	0.0296 (7)	0.0217 (6)	0.0042 (5)	0.0045 (5)	0.0029 (5)
C14	0.0214 (7)	0.0237 (7)	0.0212 (6)	0.0004 (5)	0.0080 (5)	0.0016 (5)
C15	0.0270 (7)	0.0205 (7)	0.0296 (7)	−0.0032 (5)	0.0097 (6)	0.0032 (5)

### Geometric parameters (Å, °)

**Table:**

O1—C1	1.2095 (15)	C6—C7	1.3812 (18)
O2—C8	1.2077 (15)	C6—H6	0.9500
O3—C10	1.3604 (15)	C7—C8	1.4862 (17)
O3—C15	1.4296 (16)	C9—C14	1.3818 (18)
N1—C1	1.4057 (16)	C9—C10	1.4010 (18)
N1—C8	1.4073 (16)	C10—C11	1.3946 (17)
N1—C9	1.4301 (15)	C11—C12	1.3885 (19)
C1—C2	1.4865 (17)	C11—H11	0.9500
C2—C3	1.3821 (17)	C12—C13	1.383 (2)
C2—C7	1.3910 (17)	C12—H12	0.9500
C3—C4	1.3923 (18)	C13—C14	1.3879 (18)
C3—H3	0.9500	C13—H13	0.9500
C4—C5	1.3937 (18)	C14—H14	0.9500
C4—H4	0.9500	C15—H15A	0.9800
C5—C6	1.3927 (18)	C15—H15B	0.9800
C5—H5	0.9500	C15—H15C	0.9800
			
C10—O3—C15	116.90 (10)	O2—C8—C7	129.18 (12)
C1—N1—C8	111.90 (10)	N1—C8—C7	105.46 (10)
C1—N1—C9	124.33 (10)	C14—C9—C10	120.61 (11)
C8—N1—C9	123.56 (10)	C14—C9—N1	118.93 (11)
O1—C1—N1	124.77 (12)	C10—C9—N1	120.43 (11)
O1—C1—C2	129.54 (11)	O3—C10—C11	124.71 (11)
N1—C1—C2	105.66 (10)	O3—C10—C9	116.50 (11)
C3—C2—C7	121.35 (11)	C11—C10—C9	118.79 (11)
C3—C2—C1	130.42 (11)	C12—C11—C10	119.85 (12)
C7—C2—C1	108.21 (11)	C12—C11—H11	120.1
C2—C3—C4	117.57 (12)	C10—C11—H11	120.1
C2—C3—H3	121.2	C13—C12—C11	121.12 (12)
C4—C3—H3	121.2	C13—C12—H12	119.4
C3—C4—C5	120.89 (12)	C11—C12—H12	119.4
C3—C4—H4	119.6	C12—C13—C14	119.12 (12)
C5—C4—H4	119.6	C12—C13—H13	120.4
C6—C5—C4	121.32 (12)	C14—C13—H13	120.4
C6—C5—H5	119.3	C9—C14—C13	120.42 (12)
C4—C5—H5	119.3	C9—C14—H14	119.8
C7—C6—C5	117.29 (12)	C13—C14—H14	119.8
C7—C6—H6	121.4	O3—C15—H15A	109.5
C5—C6—H6	121.4	O3—C15—H15B	109.5
C6—C7—C2	121.53 (11)	H15A—C15—H15B	109.5
C6—C7—C8	129.88 (12)	O3—C15—H15C	109.5
C2—C7—C8	108.59 (11)	H15A—C15—H15C	109.5
O2—C8—N1	125.34 (11)	H15B—C15—H15C	109.5
			
C8—N1—C1—O1	−174.23 (12)	C9—N1—C8—C7	−176.91 (11)
C9—N1—C1—O1	0.68 (19)	C6—C7—C8—O2	−1.5 (2)
C8—N1—C1—C2	3.86 (13)	C2—C7—C8—O2	177.74 (13)
C9—N1—C1—C2	178.77 (11)	C6—C7—C8—N1	179.86 (12)
O1—C1—C2—C3	−4.7 (2)	C2—C7—C8—N1	−0.90 (13)
N1—C1—C2—C3	177.31 (12)	C1—N1—C9—C14	−117.21 (13)
O1—C1—C2—C7	173.64 (12)	C8—N1—C9—C14	57.11 (17)
N1—C1—C2—C7	−4.33 (13)	C1—N1—C9—C10	64.41 (16)
C7—C2—C3—C4	1.02 (18)	C8—N1—C9—C10	−121.27 (13)
C1—C2—C3—C4	179.20 (12)	C15—O3—C10—C11	−7.96 (18)
C2—C3—C4—C5	−1.90 (18)	C15—O3—C10—C9	172.06 (11)
C3—C4—C5—C6	0.75 (19)	C14—C9—C10—O3	−177.71 (11)
C4—C5—C6—C7	1.31 (19)	N1—C9—C10—O3	0.64 (17)
C5—C6—C7—C2	−2.21 (19)	C14—C9—C10—C11	2.31 (18)
C5—C6—C7—C8	176.94 (12)	N1—C9—C10—C11	−179.34 (11)
C3—C2—C7—C6	1.07 (19)	O3—C10—C11—C12	177.10 (12)
C1—C2—C7—C6	−177.47 (11)	C9—C10—C11—C12	−2.92 (19)
C3—C2—C7—C8	−178.24 (11)	C10—C11—C12—C13	1.2 (2)
C1—C2—C7—C8	3.22 (13)	C11—C12—C13—C14	1.2 (2)
C1—N1—C8—O2	179.33 (12)	C10—C9—C14—C13	0.07 (19)
C9—N1—C8—O2	4.38 (19)	N1—C9—C14—C13	−178.31 (11)
C1—N1—C8—C7	−1.96 (13)	C12—C13—C14—C9	−1.8 (2)
